# The Etiology and Epidemiological Features of Acute Pancreatitis in Saudi Arabia: A Systematic Review

**DOI:** 10.7759/cureus.46511

**Published:** 2023-10-05

**Authors:** Ahmed A Almohammadi, Owais H Aljafri, Hossam H Esawi, Anas A Alzhrani, Muteb S Alharbi

**Affiliations:** 1 Preventive Medicine and Public Health, Saudi Commission for Health Specialties, Medina, SAU

**Keywords:** mortality, saudi arabia, etiology, epidemiology, acute pancreatitis

## Abstract

We aimed to identify the etiology of acute pancreatitis (AP) and its most common causes with its epidemiological features among the Saudi population in 2023 in different regions. In this systematic review, we assessed the etiology and epidemiological features of acute pancreatitis in Saudi Arabia following Preferred Reporting Items for Systematic Reviews and Meta-Analyses (PRISMA) 2009 guidelines. Inclusion criteria were studies conducted in Saudi Arabia, published in English, and involving participants aged 18 years or older. PubMed and Google Scholar were searched in March 2023 for English articles published between 1985 and 2023 using specific keywords. Two reviewers screened titles, abstracts, and full-text articles for eligibility, with disagreements resolved by a third reviewer. Data on study characteristics, participant demographics, and etiological factors were extracted using a standardized form. Descriptive analysis summarized the etiology of acute pancreatitis in Saudi Arabia based on the extracted data.

Out of the 58 studies retrieved for screening, 10 studies were included in the final systematic review, and most of them were done in the Riyadh region. The sum of the sample size was 1,695 participants. In Saudi Arabia, the most prevalent cause of acute pancreatitis is biliary stones. The average mortality rate of acute pancreatitis in reported studies is 2.2%. In conclusion, biliary and idiopathic causes are the most frequent etiologies of AP in some different regions of Saudi Arabia, acute peripancreatic fluid collections and pancreatic pseudocysts are the two most commonly reported complications associated with AP, and the mortality rate of AP in Saudi Arabia may be higher compared to developed countries.

## Introduction and background

Pancreatitis is a disease that attacks the pancreas, which is one of the visceral organs located behind the stomach. The pancreas produces digestive enzymes and other hormones such as insulin. The disease occurs when digestive enzymes affect the pancreas and cause inflammation [1]. This disease has two types: acute pancreatitis (AP) and chronic pancreatitis. Either form is serious and can lead to complications [2]. Acute pancreatitis (AP) is a medical emergency, and its symptoms can vary from mild form, which resolves by effective treatment within days, to severe form, which can lead to organ failure and even death [3]. AP can be classified into mild AP, where systemic complication and organ failure are absent; moderate AP, when an organ failure occurs but can be resolved with effective treatment; and severe AP, when one or multiple organ failure become persistent [3]. Globally, AP occurs annually in about 30 per 100,000 people [4]. People with certain characteristics are more vulnerable to being affected by acute or chronic pancreatitis compared with others. Males are more likely to be affected by the disease compared to females. Also, those with a personal or a family history of gallstones or having a positive family history of pancreatitis have a higher risk of being affected by AP [1].

The signs and symptoms of AP vary and may include pain in the right upper quadrant radiating to the back, nausea, and vomiting that is worsened with eating. A fever may occur, and jaundice appears when the gallbladder is affected by gallstones [1]. Bile duct obstruction by gallstones and heavy alcohol use are the two most common causes of AP. Both are responsible for around 80% of pancreatitis [5]. Direct trauma; certain medications such as statins, angiotensin-converting enzyme (ACE) inhibitors, oral contraceptives/hormone replacement therapy (HRT), diuretics, antiretroviral therapy, and oral hypoglycemic agents; infections with some types of bacteria, viruses, parasites, and fungi; smoking; and tumors are considered as causes of AP [6]. Other causes including autoimmune diseases such as cystic fibrosis, genetic disorders (pancreas divisum), high blood levels of fats, and obesity can play a role in the disease [1]. Also, recent procedures with endoscopic retrograde cholangiopancreatography (ERCP) can be a cause of AP [1]. Diabetes mellitus (DM) type 2 is associated with a 2.8-fold higher risk of AP [7]. Some reasons that are not clear (idiopathic) have become more common nowadays to cause AP [8], and although the disease is rare in children, the number of children who have the disease has grown rapidly [8].

AP complications are divided into early and late complications. Early complications include inflammation of the pancreas, diabetes mellitus, infection, bleeding, ascites (when fluid is collected in the abdominal cavity), and shock [1]. Late complications happen with recurrent attacks of pancreatitis, and it may lead to pancreatic cancer, necrosis of the pancreas with abscess, and pseudocyst formation. This occurs in approximately 3% of AP cases [9]. Diagnosis requires two of the following three criteria: acute onset of right upper quadrant or vague abdominal pain that may radiate to the back, serum amylase or lipase levels more than three times the upper limit of normal, and a characteristic change in imaging study [1]. To diagnose AP, abdominal ultrasound, computed tomography (CT) scan, and magnetic resonance imaging (MRI) can be helpful [1]. The most sensitive and specific image to diagnose AP that is caused by gallstones is an abdominal ultrasound, which is a noninvasive, simple, and inexpensive modality [5]. In 25%-35% of AP patients, the bowel gas prevents the technician from viewing the gallstone [10]. If the abdominal ultrasound is inconclusive, a CT scan can be performed [10]. In the case of suspected biliary AP, ERCP can be performed as a diagnostic and therapeutic procedure to remove the gallstone, if present [1].

The treatment of AP is supportive, and it requires admission to a general hospital ward in the case of mild AP or to the intensive care unit (ICU) in the case of severe AP. Patients must keep non-per-mouth (NPO) until the inflammation resolved, and a nasogastric tube needs to be placed in the stomach to provide nutrients and oral analgesia [1]. Intravenous fluids to control dehydration, pain medication, and sometimes antibiotics may be used. The underlying cause must be treated: cholecystectomy to remove gallstone, stopping causative medications by discontinuing alcohol and smoking, etc. [1]. Surgery may be done in the case of pancreatic necrosis to remove the affected part of the pancreas [9]. The severe form of AP is considered a bad prognostic factor. It may increase the mortality rate between 2% and 9%, and this can increase if pancreatic necrosis is present [11]. To control or prevent AP, the National Institutes of Health (NIH) advises to stop drinking alcohol as it increases the episodes of acute pancreatitis, which may lead to chronic pancreatitis and increase the risk of severe complications and even death [1]. Also, the NIH advises to stop smoking as smoking may raise pancreatic cancer risk in patients with pancreatitis [1].

Following a healthy lifestyle with weight loss, if needed, may protect the pancreas, prevent the formation of gallstones as the leading cause of AP, and reduce the chance of getting obesity and diabetes, which are risk factors for the disease [1]. AP is a medical emergency that needs a quick, effective treatment. It can affect many organs of the body and may cause organ failure and even death [2]. Previous studies suggested a steady increase in AP-related hospitalizations [6]. Lack of knowledge regarding the etiology and risk factors of AP among the Saudi population can lead to a rapid increase in disease occurrence. This may decrease the quality of life and increase medical costs and their complications. Therefore, this review aims to identify the etiology of AP and its risk factors and understand the sociodemographic characteristics of the Saudi population with AP.

## Review

Methodology

This systematic review followed the Preferred Reporting Items for Systematic Reviews and Meta-Analyses (PRISMA) 2009 guidelines. The aim of this study was to conduct a comprehensive evaluation of the etiology of acute pancreatitis in Saudi Arabia.

Inclusion Criteria

For inclusion in this review, studies had to meet the following criteria: (a) conducted in Saudi Arabia, (b) published in the English language, and (c) involved participants aged 18 years or older. A thorough literature search was conducted in PubMed and Google Scholar databases. The search strategy employed combinations of the following keywords: "acute pancreatitis," "Saudi Arabia," and "etiology." The search was conducted in March 2023, and the scope was limited to articles published in English between January 1985 and March 2023.

Two independent reviewers screened the titles and abstracts of the identified articles based on the specified inclusion criteria. Full-text articles of potentially relevant studies were then assessed for eligibility. Any disagreements were resolved through discussion or consultation with a third reviewer. A standardized data extraction form was used to extract pertinent information from the included studies. This encompassed study characteristics (author, year, and type of study), participant demographics (age, gender, and region), and etiological factors associated with acute pancreatitis. The quality of the included studies was assessed using the Joanna Briggs Institute (JBI) Critical Appraisal Tools, which are appropriate quality assessment tools for this purpose. Two independent reviewers evaluated the risk of bias within each study, and any discrepancies that arose were resolved through comprehensive discussions or by seeking consultation with a third reviewer. Descriptive analysis was employed to summarize the etiology of acute pancreatitis in Saudi Arabia, based on the extracted data.

Result

**Figure 1:**
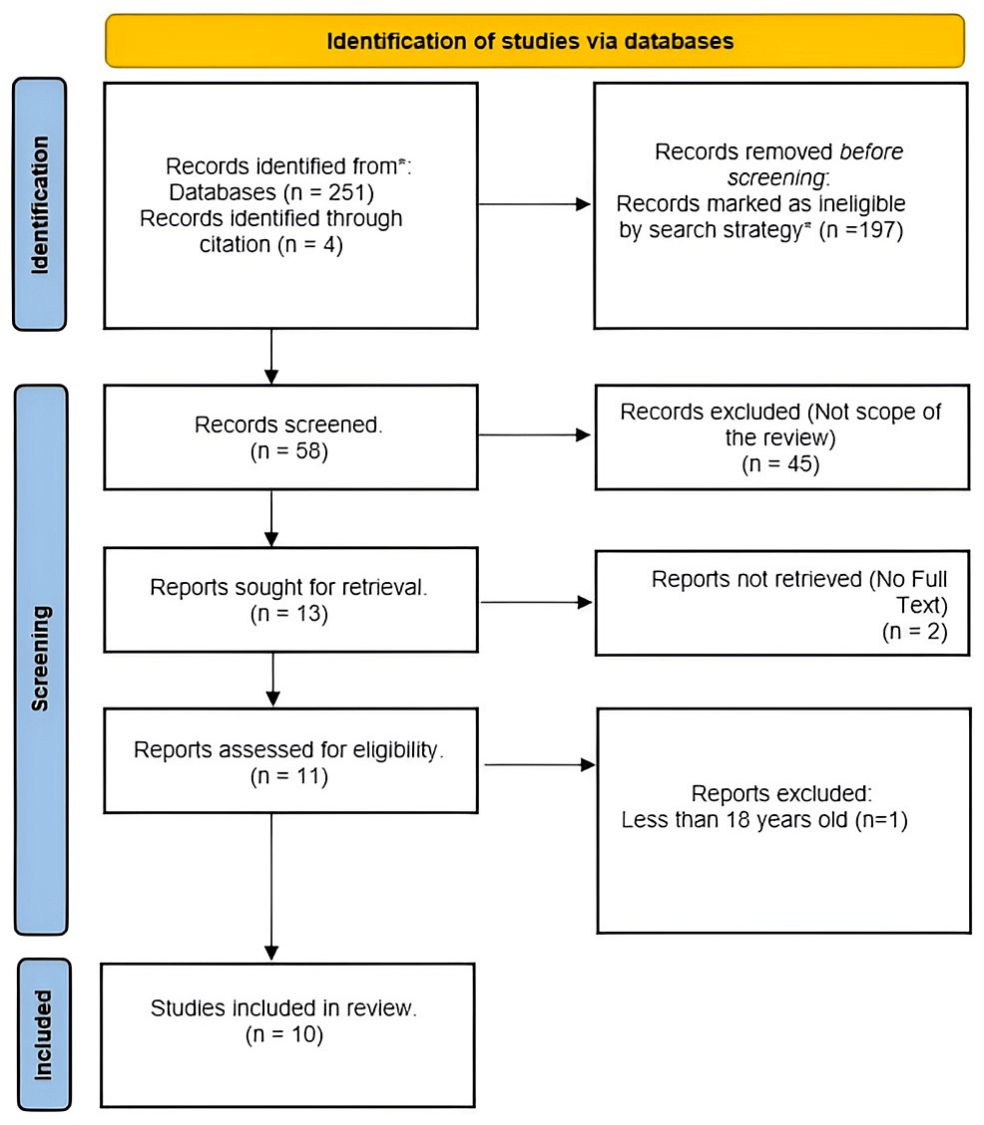
PRISMA 2020 flow diagram PRISMA: Preferred Reporting Items for Systematic Reviews and Meta-Analyses

Out of the 255 studies retrieved from databases and reference searching, our search strategy limited the records to be screened to 58 records. However, only 10 studies were included in our final review; other studies did not meet our inclusion criteria or were unavailable for retrieval (Figure 1).

**Figure 2:**
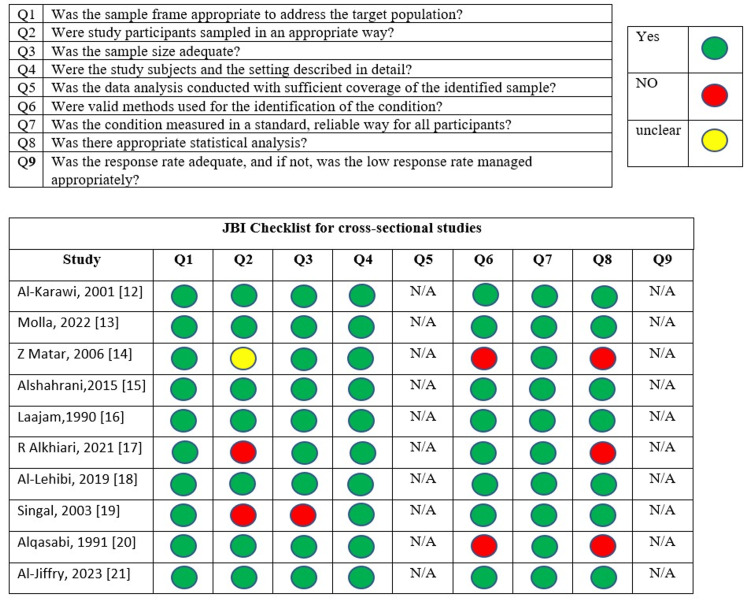
Quality assessment of all the included studies using the JBI Critical Appraisal Tools JBI: Joanna Briggs Institute References: [12-21]

The quality of the included studies was assessed using the JBI Critical Appraisal Tools checklist (Figure 2).

These studies were conducted between 1985 and 2023. All included studies were conducted in Saudi Arabia and took place in three different regions. However, most of them happened within the Riyadh region, six studies to be exact. All the retrieved research was retrospective in design, except one study done by Al-Qasabi et al. [20], which was labeled as a prospective study.

Table 1 shows a summary of the 10 studies emphasizing each study participation size of 1,695 in total between all studies.

**Table 1:** Summary of the included studies The table shows the results of studies on the mean (median) age and sex ratio of patients with AP in Saudi Arabia.

First author	Study period	Region	Sample size	Mean/median age	Sex ratio (male percent)
Al-Karawi (2001) [12]	1985-1997	Riyadh	218	37 years (median)	44.04%
Molla (2022) [13]	2008-2021	Riyadh	327	45 years (median)	56.4%
Matar (2006) [14]	1993-1999	Al-Kharaj	96	43 years (mean)	42%
Alshahrani (2015) [15]	2012-2014	Bisha	544	46 years (median)	21%
Laajam (1990) [16]	1984-1988	Riyadh	104	42.4 years (mean)	34%
Alkhiari (2021) [17]	2017-2019	Buraydah	37	48.7 years (median)	59.46%
Al Lehibi (2019) [18]	2014-2017	Riyadh	107	48 years (mean)	53%
Singal (2003) [19]	1990-2001	Jazan	71	42.6 years (mean)	41.9%
Al-Qasabi (1991) [20]	1986-1989	Riyadh	124	46.5 years (mean)	53%
Aljiffry (2023) [21]	2017-2021	Jeddah	67	48.9 years (mean)	44.8%

Our review revealed that the most commonly reported etiology for acute pancreatitis in Saudi Arabia is biliary in nature. Eight of the studies showed biliary stone or sludge as the most frequent cause. However, two of the included studies described the most common cause as idiopathic.

Table 2 shows a summary of the causes of acute pancreatitis and their percentage in each study.

**Table 2:** Summary of causes in each study

Study	Biliary	Idiopathic	Alcohol	Postoperative	Hyperlipidemia	Infection	Drugs	Trauma	Autoimmune	Other
Al-Karawi (2001) [12]	67.5%	17%	1.8%	5.9%	3.2%	1.8%	-	-	-	1.8% (rare causes)
Molla (2022) [13]	15.6%	76.8%	-	4.3%	-	-	-	-	-	-
Matar (2006) [14]	50%	38.5%	3.13%	-	7.29%	-	-	1.04%	-	-
Alshahrani (2015) [15]	81%	4%	7%	2%	-	3%	2%	-	-	1% (cancer)
Laajam (1990) [16]	67.3%	11.5%	6.7%	1%	6.7%	-	2.9	2%	-	2% (uremic)
Alkhiari (2021) [17]	40.5%	46%	5.4%	-	-	2.7%	-	2.7%	2.7%	-
Al Lehibi (2019) [18]	39.3%	15%	11.2%	4.7%	8%	-	2.8%	1.9%	1.9%	7.5% (multiple), 2.8% (cancer)
Singal (2003) [19]	41.9%	25.8%	-	17.7%	1.6%	3.2%	4.8%	-	3.2%	1.6 (cancer)
Al-Qasabi (1991) [20]	54%	24%	7%	6%	1%	-	5%	1%	1%	1% (burns)
Aljiffry (2023) [21]	55.2%	25.4%	6%	-	-	-	-	-	10.4%	3% (iatrogenic)

Moreover, six of the reviewed studies included mortality statistics in their reports; most of them showed less than a 5% mortality rate. Table 3 shows the mortality data and a summary of the outcomes reported in each study.

**Table 3:** Severity of cases, reported complications, and mortality rate in each study APFC: acute peripancreatic fluid collections, ICU: intensive care unit, DM: diabetes mellitus, BISAB: bedside index for severity in acute pancreatitis, GI: gastrointestinal

Study	Percentage of severe cases	Criteria used	Complications reported	Mortality rate
Al-Karawi (2001) [12]	32%	Modified Glasgow (Imrie)	-	1.83%
Molla (2022) [13]	-	-	APFC in 48.3%	-
Matar (2006) [14]	34.3%	Ranson's criteria	Pancreatic pseudocysts: 5%, pleural effusion: 3.1%, DM: 2.1%, pneumonia: 1%, pancreatic fistula: 1%, acute nephritic syndrome: 1%	-
Alshahrani (2015) [15]	5%	Unknown	5% ICU admission	0.4%
Laajam (1990) [16]	21%	Modified Glasgow (Imrie)	Pancreatic pseudocysts: 6.6%, pancreatic abscess: 1%	3.3%
Alkhiari (2021) [17]	46% (moderate)	Atlanta Classification	APFC: 24.3%, pancreatic necrosis: 13.5%, thickening of GI wall, pleural effusion: 13.5%	-
Al Lehibi (2019) [18]	-	-	Pancreatic pseudocysts: 15%, APFC: 9%, necrosis: 3.7%	7.5%
Singal (2003) [19]	11.3%	Ranson's criteria	-	4.8%
Al-Qasabi (1991) [20]	33.9%	Ranson's criteria	-	5.3%
Aljiffry (2023) [21]	10.45%	BISAB	APFC: 25.4%, atelectasis: 14.9%, pleural effusion: 9%, ascites or pseudocysts: 6%, necrosis: 3.7%, pancreatic abscess: 1.5%	-

Discussion

In our review, most of the studies done had a higher female sex ratio than male. Almost all the study participants' age falls between 40 and 50 years. Eight of the studies showed biliary etiology as the most common cause of AP, followed by idiopathic etiology as the second most common cause. Two studies that have been conducted in Riyadh and Buraydah region showed idiopathic etiology as the most common cause of AP, followed by biliary etiology as the second most common cause. All studies reported other possible and less common etiologies for AP in different regions, including alcohol, postoperative, hyperlipidemia, infections, drugs, trauma, and autoimmune causes.

Furthermore, six out of the 10 studies reported the percentage of the case severity level of participants, with the Al-Kharaj region having the highest reported percentage of severe cases, while the Bisha region had the lowest percentage of severe cases, although the criteria used in this study are unknown. A single study done in the Buraydah region reported only moderate severity cases. Some of the studies reported complications, with acute peripancreatic fluid collections and pancreatic pseudocysts being the two most common complications associated with AP in different regions. The lowest mortality rate was reported in the Bisha region (0.4%), while the highest mortality rate was reported in the Riyadh region (7.5%).

In developed countries, obstruction of the common bile duct by stones (38%) and alcohol abuse (36%) are the most frequent causes of AP [22]. Our review revealed that biliary and idiopathic causes are the most frequent etiologies of AP in some different regions of Saudi Arabia. This may be because alcohol use is less common in Arabic countries, and it is even prohibited in Saudi Arabia [23]. Mortality from acute pancreatitis decreased from 12% to 2%, according to a large epidemiologic study from the United States [24], compared to our review which revealed a higher mortality rate in multiple studies in some regions.

Some limitations in our review were the lack of relevant published papers in many other regions in Saudi Arabia, as the studies included were done in only three different regions. In addition, not all the studies reported complications, the severity level of cases, or the mortality rate. Many studies lacked other contextual data since the variables collected were not enough to provide a complete risk factor profile in our review, which in total may have influenced the overall result, and the findings may not be consistent in some other regions. Further studies should be conducted in different regions for a better understanding of the AP epidemiology profile in Saudi Arabia, as this may help in the prevention of the common etiologies and complications of AP to reduce incidence and mortality rates.

## Conclusions

Our review found that biliary and idiopathic causes are the most frequent etiologies of AP in different regions of Saudi Arabia, with a higher female sex ratio, and most participants aged between 40 and 50 years. Acute peripancreatic fluid collections and pancreatic pseudocysts were the two most commonly reported complications associated with AP. The mortality rate was highest in the Riyadh region (7.5%) and lowest in the Bisha region (0.4%). The mortality rate of AP in Saudi Arabia may be higher compared to developed countries. We recommend further studies to be conducted in a prospective manner at a national level of multiple regions for a better understanding of AP epidemiology patterns in Saudi Arabia as this may help in the prevention of the common etiologies and complications of AP to reduce incidence and mortality rates.
